# *Nigella sativa* and Its Protective Role in Oxidative Stress and Hypertension

**DOI:** 10.1155/2013/120732

**Published:** 2013-03-07

**Authors:** Xin-Fang Leong, Mohd Rais Mustafa, Kamsiah Jaarin

**Affiliations:** ^1^Department of Pharmacology, Faculty of Medicine, Universiti Kebangsaan Malaysia, Jalan Raja Muda Abdul Aziz, 50300 Kuala Lumpur, Malaysia; ^2^Department of Clinical Oral Biology (Pharmacology), Faculty of Dentistry, Universiti Kebangsaan Malaysia, Jalan Raja Muda Abdul Aziz, 50300 Kuala Lumpur, Malaysia; ^3^Department of Pharmacology, Faculty of Medicine, University of Malaya, 50603 Kuala Lumpur, Malaysia

## Abstract

Hypertension increases the risk for a variety of cardiovascular diseases, including stroke, coronary artery disease, heart failure, and peripheral vascular disease. The increase in oxidative stress has been associated with the pathogenesis of hypertension. Increase of blood pressure is due to an imbalance between antioxidants defence mechanisms and free radical productions. Excessive production of reactive oxygen species reduces nitric oxide bioavailability leading to an endothelial dysfunction and a subsequent increase in total peripheral resistance. Hypertension can cause few symptoms until it reaches the advanced stage and poses serious health problems with lifelong consequences. Hypertensive patients are required to take drugs for life to control the hypertension and prevent complications. Some of these drugs are expensive and may have adverse reactions. Hence, it is timely to examine scientifically, complimentary therapies that are more effective and with minimal undesirable effects. *Nigella sativa* (NS) and its active constituents have been documented to exhibit antioxidant, hypotensive, calcium channel blockade and diuretic properties which may contribute to reduce blood pressure. This suggests a potential role of NS in the management of hypertension, and thus more studies should be conducted to evaluate its effectiveness.

## 1. Introduction

According to The Seventh Report of the Joint National Committee on Prevention, Detection, Evaluation and Treatment of High Blood Pressure, hypertension is diagnosed as systolic blood pressure (BP) which is greater than 140 mmHg and/or diastolic BP which is greater than 90 mmHg [1]. The prevalence of hypertension in most developing countries is comparable to the developed countries [2, 3]. Hypertension is a major global health disorder due to prolonged human life span. Familial influence and environmental factors such as obesity, sedentary life style, and unhealthy dietary habit contribute to the high prevalence of hypertension [4–6]. The prevalence of hypertension in Malaysians aged 30 years and above was 42.6% in year 2006, a relative increase of 30% compared to 10 years earlier [7]. In the year 2000, it was estimated about 972 million world's adult population had hypertension. This number will increase to 1.56 billion by the year 2025 [8].

In recent years, there has been a growing interest and demand in using medicinal plants for treating and preventing various diseases including cardiovascular diseases. Traditional medicines of plants origin have received much attention due to several factors such as easy availability, affordable cost, safety, and efficacy as well as cultural acceptability. *Nigella sativa *(NS), or also known as black cumin or its Arabic name *habat-ul sauda*, has been used for centuries in medicinal and culinary purposes throughout the Middle East, India, and Northern Africa. It is an annual flowering plant with pale blue flowers that belongs to the Ranunculaceae family. The plant has a fruit which contains angular black seeds, and the seeds are considered to be the most valuable part contributing beneficial health effects. NS as a natural remedy has been documented to possess numerous therapeutic values, including diabetes, tumour, hypercholesterolemia, hypertension, inflammation, and gastrointestinal disorders [9–14]. The present paper is, therefore, to examine the current literature on the cardiovascular protective effects of NS and its constituents against oxidative stress and hypertension in addition to the possible mechanisms of actions underlying these beneficial effects.

## 2. *Nigella sativa* (NS)

The seed oil of NS was found to be rich in polyphenols and tocopherols [25, 26]. The seeds contain 36–38% fixed oils, 0.4–2.5% essential (volatile) oil, proteins, alkaloids, and saponins [27]. The fixed oil is composed mainly of fatty acids, namely, linoleic (C18:2), oleic (C18:1), palmitic (C16:0), and stearic (C18:0) acids [28]. Thymoquinone (TQ) is the most pharmacologically active ingredient found abundantly (30–48%) in the black seeds, together with its derivatives such as dithymoquinone, thymohydroquinone, and thymol [29].

## 3. Antioxidant Property of NS

The seed oil of NS is well known for its strong antioxidant properties [30]. Previous studies have documented that pre-treatment with TQ, the main active constituents in seed oil, protected organs against oxidative damage induced by a variety of free radical generating agents, such as carbon tetrachloride [31] and including the alkylating agents, cisplatin [32], and doxorubicin [33]. The free radical scavenging effects of TQ, dithymoquinone, and thymol were tested against several reactive oxygen species (ROS) [34]. All the tested compounds from NS exerted strong antioxidant effects; thymol acted as singlet oxygen quencher, while TQ and dithymoquinone showed superoxide dismutase (SOD)-like activity [34]. In addition, a study carried out by Mansour et al. [35] revealed that both TQ and dithymoquinone acted not only as superoxide anion scavengers, but also as general free radical scavengers with half maximal inhibitory concentration (IC_50_) in the nanomolar and micromolar ranges, respectively. These findings suggest the importance of such free radical scavenging compounds in the treatment of hypertension which is closely associated with oxidative stress.

TQ is a potent superoxide radical scavenger which is as effective as SOD against superoxides generated either photochemically, biochemically, or derived from calcium ionophore (A23817) [36]. Furthermore, TQ has an inhibitory effect on lipid peroxidation induced by Fe^3+^/ascorbate. In rats, TQ is protective against doxorubicin-induced cardiotoxicity by reducing the elevation of serum lactate dehydrogenase and creatine phosphokinase levels [36]. Ismail et al. [37] showed that both TQ-rich fraction and TQ markedly improved plasma antioxidant status by inhibiting formation of hydroxyl radicals. Moreover, liver antioxidant enzymes (SOD and glutathione peroxidase GPx) are significantly increased in rats treated with TQ-rich fraction and TQ. In the same study, both TQ-rich fraction and TQ caused an enhanced expression of antioxidant genes (SOD-1, catalase CAT, and GPx-2) in hypercholesterolemic rats [37].

Erşahin et al. [38] reported that NS oil with its potent free radical scavenging properties, inhibited subarachnoid-haemorrhage-(SAH-) induced lipid peroxidation of the brain tissue in rat against the reactive hydroxyl, peroxyl, and superoxide radicals. In addition, the level of antioxidant glutathione (GSH) was preserved [38], thereby ameliorating oxidative damage. The SAH-induced reduction of Na^+^/K^+^-ATPase activity indicated the presence of membrane damage. The Na^+^/K^+^-ATPase is involved in the generation of the membrane potential through the active transport of sodium and potassium ions in cellular membrane. It maintains neuronal excitability and controls cellular volume in the central nervous system. Treatment with NS oil was able to restore Na^+^/K^+^-ATPase activity back to normal levels [38].

Administration of TQ restored the activities of nonenzymatic (GSH and vitamin C) and enzymatic (SOD, CAT, GPx, and glutathione-S-transferase GST) antioxidants as well as reduced the levels of malondialdehyde (MDA) in the rat brain to normal levels [39]. Besides that, TQ supplementation resulted in a complete reversal of the gentamicin-(GM-) induced increase in blood urea nitrogen, creatinine, thiobarbituric acid reactive substances (TBARS), and nitric oxide (NO) and decrease in GSH, GPx, CAT, and adenosine triphosphate (ATP) to control values [40]. Histopathological examination of kidney tissues was in agreement with the biochemical data, wherein TQ supplementation prevented GM-induced degenerative changes in kidney tissues [40]. The findings from this study demonstrated the strong protective effect of TQ by its ability to decrease oxidative stress and to preserve the activities of the antioxidant enzymes [40].

## 4. Pathogenesis of Hypertension

### 4.1. Role of Oxidative Stress in Hypertension

Free radicals possess one or more unpaired electrons in their outer electronic orbits. ROS such as superoxide anion (O_2_^−^), hydroxyl radical (OH^−^), hydrogen peroxide (H_2_O_2_), and singlet oxygen (^1^O_2_) are highly reactive. Free radicals and ROS are formed continuously in normal physiological process [41]. However, excessive production leads to oxidative stress with an increase in the formation of nitrogen-oxygen derivative free radicals as well as a decrease in antioxidant capacity [42].

Oxidative stress occurs when there is an imbalance between production of ROS and antioxidant defence system in favour of the former [43, 44]. ROS are highly reactive and unstable by nature; hence, they can damage various cellular components including lipid membranes. Lipid peroxides are derived from polyunsaturated fatty acid (PUFA) oxidation and are capable to initiate lipid peroxidation via free radicals chain reaction. MDA is a major end product of PUFA peroxidation and is often used as an indicator of cell injury.

Increase in the production of MDA may be due to the formation of reactive oxidants. Lipid peroxidation leads to structural changes of the lipid molecules, and the changes are more severe as lipids are the main constituent of biological membranes. Generally, lipid peroxides pose a risk factor for atherosclerotic complications. Increase in free radical formation is linked to a reduction in NO generation [45]. Elevation in serum MDA level found in hypertensive patients suggests a relationship with the increased oxidative stress [46].

Endothelium-derived relaxing factor (EDRF) or better known as NO plays an important regulatory role in the maintenance of vascular homeostasis. BP is regulated by cardiac output and peripheral resistance of blood vessels. NO causes vasodilatation, subsequently reducing total peripheral resistance. Endothelial dysfunction is associated with abnormal endothelium-dependent relaxation as observed in hypertension [47–49]. Reduced NO bioavailability, that is, a reduction in NO production by free radicals or an increase in deactivation of NO due to imbalance between antioxidant and oxidant levels may be the mechanism underlying endothelium dysfunction.

NO is synthesized predominantly in the vascular endothelium. Endothelial nitric oxide synthase (eNOS) is required for the synthesis of NO from amino acid L-arginine. There are two other isoforms of NOS, namely, neuronal nitric oxide synthase (nNOS) and inducible nitric oxide synthase (iNOS). Both eNOS and nNOS are constitutive enzymes that are produced in normal physiological process, while iNOS is mainly induced during inflammation. NO is small in size and lipophilic by nature. This allows NO to diffuse rapidly through cell membranes to the adjacent smooth muscle cells. Eventually, NO activates guanylate cyclase (GC), and stimulates the formation of cyclic guanosine monophosphate (cGMP) that leads to vascular smooth muscle relaxation. Generation of ROS such as superoxide anions may cause cellular injury by oxidizing membrane lipids, proteins, and nucleic acids. Furthermore, superoxide anions may reduce NO bioavailability by binding to the gaseous molecule and forming peroxynitrite which itself is a free radical [50].

Angiotensin-converting enzyme (ACE) plays a vital role in the regulation of BP and electrolytes balance. ACE is mainly located on the surface of endothelium and epithelium. It hydrolyses angiotensin I (Ang I) to angiotensin II (Ang II), a potent vasoconstrictor and aldosterone-stimulating peptide. Ang II is an important factor in cardiovascular homeostasis [51]. The importance of ACE in maintaining the BP can be observed via the beneficial effect of ACE inhibitors in treating hypertension [52, 53]. Ang II induces oxidative stress via activation of nicotinamide adenine dinucleotide/nicotinamide adenine dinucleotide phosphate (NADH/NADPH) oxidase and the production of ROS [54]. In addition to that, Ang II increases lipid peroxidation [55] and stimulates the production of prooxidant cytokines [56, 57], in which these subsequently lead to elevation of BP. Ang II decreases NOS expression and stimulates the generation of ROS [54].

Ang II-induced hypertension is associated with increased vascular superoxide production and impaired vasorelaxation to acetylcholine [54]. Ang II exacerbates oxidative stress, and the increase in the superoxide level could result in endothelial dysfunction via scavenging NO and decreasing NO bioavailability [58]. Hence, the NO-Ang II imbalance may be an important component in the vascular pathophysiology of hypertension. Ang II has been suggested to cause an increase in oxidized low-density lipoprotein cholesterol (ox-LDL) uptake, eventually causing endothelial cell injury [59]. On the other hand, it has also been suggested that ox-LDL upregulates Ang II type 1 receptor expression [60]. These observations may indicate the presence of a relationship between ox-LDL and rennin-angiotensin systems in hypertension. Hence, these two systems may be responsible for the development of endothelial dysfunction, leading to an increase in BP.

### 4.2. Evidences of Oxidative Stress Involved in Hypertension

There has been a growth evidence suggesting that hypertension may be attributable to the increased production of ROS [61, 62]. Oxidative stress may play a vital role in the development of hypertension via the following mechanisms: enhanced sequestration of NO by ROS [63], formation of lipid peroxidation products [64], and depletion of NOS cofactor (tetrahydrobiopterin) [65]. Lastly, it may cause functional and structural changes in vascular wall and blood vessel [66]. These vascular alterations may be mediated by several ways, including direct injury to endothelial and vascular smooth muscle cells, effects on endothelial cell eicosanoid metabolism, altered redox state, increases in intracellular free calcium concentration, stimulation of inflammatory, and growth signalling events [67, 68].

Oxidative stress promotes proliferation of vascular smooth muscle cells as well as collagen deposition which cause thickening of tunica media and narrowing of the vascular lumen [69], which eventually leads to an increase in the total peripheral resistance. Furthermore, increase in oxidative stress may damage the endothelium and impair endothelium-dependent vasodilatation, consequently increasing vascular contractile activity [69]. ROS may also induce endothelial permeability with the recruitment of inflammatory protein and cells, in which can compromise endothelial function and worsen vascular damage [70]. All these observed effects on the vasculature further support the pathological role of oxidative stress in hypertension.

Amanullah et al. [70] reported that higher MDA levels and lower antioxidant activities such as SOD, GSH, and GPx in hypertensive subjects may be due to increased production of free radicals, indicating the presence of oxidative stress in hypertension. There were notable a positive correlation between BP and lipid peroxidation products in the hypertensives and a negative correlations between BP with plasma antioxidant capacity, plasma vitamin C levels, erythrocyte activity of antioxidant enzymes, and erythrocyte reduced/oxidized glutathione (GSH/GSSG) ratio [71]. These findings demonstrated a possible role of oxidative stress in the pathophysiology of hypertension. In another study, hypertensive patients exhibited higher plasma lipid peroxides along with decreased nonenzymatic antioxidant levels, which could be associated with oxidative stress and depleted antioxidant defence potential [72]. Oxidative stress may occur due to a reduction in antioxidant activities or due to an elevation in ROS concentration. This can lead to oxidative damage to the structure of biomolecules which mostly involve the antioxidant enzymes, thus contributing to the oxidative stress in hypertensives instead of normotensive subjects.

A variety of antioxidant treatments ameliorate hypertension in animal and human studies. Veratric acid, a phenolic acid, was found to decrease the BP, significantly restored NO, enzymatic and nonenzymatic antioxidants, and reduced lipid peroxidation products against L-NG-nitroarginine methyl ester-(L-NAME-) induced hypertension in Wistar rats [73]. Park et al. [74] demonstrated that soy isoflavone supplementation elevated serum NO and total radical trapping antioxidant potential (TRAP) with a reduction in systolic BP after 30 days of feeding period to spontaneously hypertensive rats (SHRs). The results suggest that protective effect of isoflavone against hypertension occurs possibly via the mitigation of oxidative stress and augmentation of NO production. Consumption of green tea which possess strong antioxidant polyphenols was able to reduce BP, serum tumour necrosis factor-*α*, c-reactive protein, and triglycerides, and total and low-density lipoprotein cholesterol while increasing total antioxidant status and high-density lipoprotein cholesterol in patients with obesity-related hypertension [75].

Administration of vitamins C and E for eight weeks in patients with essential hypertension (EH) had significantly lowered systolic BP, diastolic BP, and mean arterial pressure compared to placebo [76]. The BP reduction was associated with higher erythrocyte and serum antioxidant capacity. BP correlated positively with plasma 8-isoprostane levels and negatively with ferric reducing ability of plasma (FRAP) levels in the vitamins C and E and placebo-treated groups [76]. The findings support the view that oxidative stress is involved in the pathogenesis of EH and that enhancement of antioxidant status by supplementation with vitamins C and E in patients with EH is associated with reduced BP. Hence, this suggests that intervention with antioxidants is a potential adjunct therapy for hypertension.

## 5. Possible Pharmacological Actions of NS against Hypertension

The exact mechanism on how NS reduces BP is not exactly known. The antihypertensive effects of NS may be due to the many active compounds, each with distinct mechanisms of actions. There are several possible mechanisms involved in BP reduction, which include cardiac depressant effect, calcium channels blocking property, and diuretic effect.  Table 1 summarized the effects of NS and its active compounds on BP.

### 5.1. Cardiac Depressant

Previous studies reported that the volatile oil and TQ decreased both the arterial BP and heart rate [15, 16]. The effects of NS on BP and heart rate were reversed by cyproheptadine (a nonselective serotonin receptor blocker) and atropine (antimuscarinic M_2_ agent) [15, 16]. This finding suggests that the protective effects of NS were mainly mediated centrally either directly or indirectly via mechanisms involving serotoninergic and muscarinic receptors [15, 16]. In contrary, El-Tahir et al. [17] documented that de-thymoquinonated volatile oil, *α*-pinene, and p-cymene from NS reduced BP and heart rate. However, hypotensive effect of NS was not reversed by atropine or cyproheptadine although these two drugs antagonized the effect of NS on heart rate [17].

The cardiac depressant effects of NS in the rats were significantly reversed by hexamethonium (a ganglionic blocker) suggesting a mechanism involving nicotinic receptors [17]. In addition, destruction of connection between the vasomotor centre in the medulla and preganglionic sympathetic by spinal pithing prevented NS-induced cardiovascular changes [17]. Therefore, the cardiac depressant and hypotensive effects of NS appeared to be mediated via central mechanism involving vasomotor centre in the medulla and sympathetic outflow to the periphery.

Failure of indomethacin (prostaglandin cyclooxygenase inhibitor), mepacrine and hydrocortisone (phospholipase A_2_ inhibitor), mepyramine (histamine H_1_ receptor blocker), ranitidine (histamine H_2_ receptor blocker), and methylene blue (NO-cGMP formation antagonist) to affect NS-induced cardiovascular depressant effects suggests a lack of involvement of eicosanoid, histaminergic, platelet-activating factor (PAF), and NO-induced mechanisms [17].

### 5.2. Calcium Channel Blockade

Thymol, another active compound of NS, has been documented to be able to reduce BP via its action on calcium (Ca^2+^) ion channels. Peixoto-Neves et al. [18] reported that thymol produced dose-dependent relaxation in rat isolated aorta. The NS-induced endothelium-independent relaxation may be mediated via mechanisms involving inhibition of Ca^2+^ release from sarcoplasmic reticulum, reduced Ca^2+^ sensitivity of the contractile system, and/or blockade of Ca^2+^ influx across the membrane [18]. Thymol induced a dose-dependent negative inotropic action on both canine and guinea-pig isolated cardiac preparations [19]. The observed effect may be due to a decrease in Ca^2+^ content in sarcoplasmic reticulum via inhibition of Ca^2+^ channel [19]. Effect of thymol on Ca^2+^ current in human and canine ventricular cardiomyocytes was investigated. Magyar et al. [77] demonstrated that thymol inhibits L-type Ca^2+^ current in a dose-dependent manner. When Ca^2+^ channels are blocked, Ca^2+^ entry into vascular smooth muscles is reduced eventually leading to an increase in vasorelaxation.

### 5.3. Diuretic

The kidney plays a vital role in the control of BP and in the pathogenesis of hypertension. Zaoui et al. [20] reported that 0.6 mL/kg of NS extract for 15 days caused 16% increase in diuresis in SHRs. The diuretic effect of NS was comparable to 5 mg/kg of furosemide which is a high ceiling diuretic. The diuretic effect was associated with an increase in urinary excretion of Na^+^, K^+^, Cl^−^, and urea [20]. This suggests that NS may decrease BP via its diuretic action. A reduction in electrolytes and water content leading to decrease in blood volume, which subsequently reducing cardiac output, is one of the main determinants for BP regulation. In another study, NS extract also demonstrated similar results with an increase in glomerular filtration rate, urinary, and electrolyte output [21]. Rennin-angiotensin-aldosterone (RAA) systems may contribute in regulating BP by controlling blood volume and peripheral vascular resistance. However, the observed effects of NS extract neither have influence on plasma ACE nor rennin activities of SHRs after 20 days of treatment [21]. Therefore, antihypertensive action of NS seems to be independent of RAA system. Nevertheless, more studies need to be performed to evaluate this hypothesis.

## 6. Conclusion

**Figure 1:**
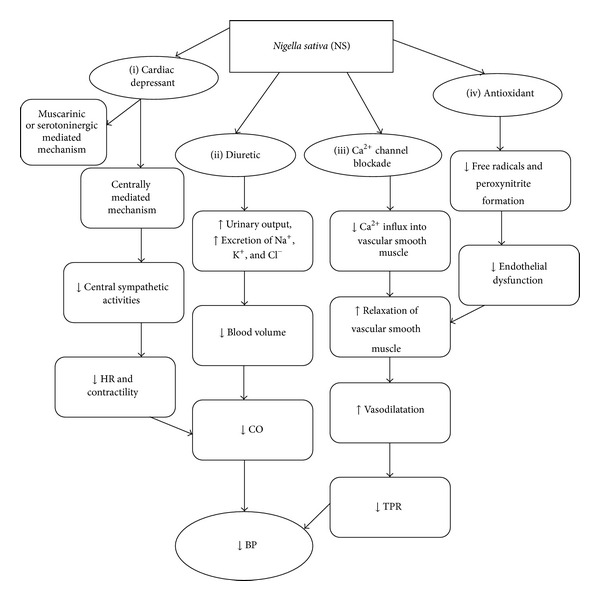
Proposed pathways for *Nigella sativa* (NS) in reducing blood pressure (BP). Ca^2+^, calcium (II) ion; Na^+^, sodium ion; K^+^, potassium ion; Cl^−^, chloride ion; HR, heart rate; CO, cardiac output; TPR, total peripheral resistance.

The cardiovascular protective effects of NS in hypertension are possibly contributed by its multitude actions including cardiac depressant, diuretic, calcium channel blockade, and antioxidant properties (Figure 1). NS is a promising medicinal plant with many therapeutic properties. Various studies have documented the protective effects of NS on the cardiovascular system against the damaging effects of various ROS, protecting the heart from cardiotoxicity as well as reducing adverse effects due to ROS involved in hypertension. NS has been used as a traditional medicine for the treatment of hypertension for many years with no report of adverse events. Further studies should be carried out on human to confirm its efficacy. It is an important area for further research and development to combine NS with other antihypertensive drugs to investigate their possible synergistic effects and preferable pharmacological properties.

## Figures and Tables

**Table 1:** Significant cardiovascular effects of NS and its constituents.

Reference	Study model	Constituents	Laboratory findings
[12]	Renovascular hypertensive rat	NS oil (i.p.) 0.2 mL/kg	↓ SBP, tissue MDA, luminol, and lucigenin CL ↑ tissue Na^+^ and K^+^-ATPase ↓ plasma CK, LDH, and ADMA ↑ plasma NO

[15]	Rat	(a) NS oil (i.v.) 4–32 *μ*L/kg (b) TQ (i.v.) 0.2–1.6 mg/kg	↓ arterial BP and heart rate (dose dependent)

[16]	Guinea pig	NS oil (i.v.) 4–32 *μ*L/kg	↓ arterial BP and heart rate (dose dependent)

[17]	Rat	(a) De-TQ volatile oil (i.v.) 2–16 *μ*L/kg (b) *α*-pinene (i.v.) 1–4 *μ*L/kg (c) p-cymene (i.v.) 2–16 *μ*L/kg	↓ arterial BP and heart rate (dose dependent) *De-TQ volatile oil and p-cymene: 4, 8, and 16 *μ*L/kg *α-pinene: 2 and 4 *μ*L/kg

[18]	Rat	Thymol (*in vitro*)	↓ aortic contraction (dose dependent)

[19]	Canine and guinea pig	Thymol (*in vitro*)	Negative inotropic action (dose dependent)

[20]	Spontaneously hypertensive rat	NS seed extract (p.o.) 0.6 mL/kg	↑ diuresis ↓ arterial BP

[21]	Spontaneously hypertensive rat	NS extract (p.o.)	↓ SBP ↑ GFR, urinary and electrolyte output

[22]	L-NAME-induced hypertensive rat	TQ (p.o.) 0.5 mg/kg and 1 mg/kg	↓ SBP and serum creatinine ↑ kidney GSH

[23]	L-NAME-induced hypertensive rat	NS seed extract (p.o.) 400 mg/kg	↓ arterial BP, SBP, DBP, and serum LDH ↑ serum NO

[24]	Patients with mild hypertension	NS seed extract (p.o.) 100 mg/kg and 200 mg/kg	↓ SBP and DBP (dose dependent) ↓ total and LDL cholesterol

NS: *Nigella sativa*; L-NAME: L-NG-nitroarginine methyl ester; i.p.: intraperitoneal; i.v.: intravenous; p.o.: per os; TQ: thymoquinone; De-TQ: de-thymoquinonated; SBP: systolic blood pressure; DBP: diastolic blood pressure; MDA: malondialdehyde; CL: chemiluminescence; CK: creatine kinase; LDH: lactate dehydrogenase; ADMA: asymmetric dimethylarginine; NO: nitric oxide; GFR: glomerular filtration rate; GSH: glutathione; LDL: low-density lipoprotein.
